# Shoulder ultrasonography performed by orthopedic surgeons increases efficiency in diagnosis of rotator cuff tears

**DOI:** 10.1186/s13018-017-0565-4

**Published:** 2017-04-20

**Authors:** Chih-Hao Chiu, Poyu Chen, Alvin Chao-Yu Chen, Kuo-Yao Hsu, Shih-Sheng Chang, Yi-Sheng Chan, Yeung-Jen Chen

**Affiliations:** 1 Department of Orthopedic Surgery, Taoyuan Chang Gung Memorial Hospital, Taoyuan, Taiwan; 2 Department of Orthopedic Surgery, Linkou Chang Gung Memorial Hospital, Taoyuan, Taiwan; 3 Musculoskeletal Research Center, Chang Gung Memorial Hospital, Linkou, Taiwan; 4 Department of Occupational Therapy and Graduate Institute of Behavioral Science, Chang Gung University, Taoyuan, Taiwan

**Keywords:** Shoulder, Ultrasonography, Rotator cuff, Efficiency, Diagnosis

## Abstract

**Background:**

Rotator cuff tears are very common and their incidence increases with age. Shoulder ultrasonography has recently gained popularity in detecting rotator cuff tears because of its efficiency, cost-effectiveness, time-saving, and real-time nature of the procedure. Well-trained orthopedic surgeons may utilize shoulder ultrasonography to diagnose rotator cuff tears. The wait time of patients planned to have shoulder MRI (magnetic resonance imaging) to rule in rotator cuff tears may decrease after orthopedic surgeon start doing shoulder ultrasonography as a screening tool for that. Patients with rotator cuff tears may be detected earlier by ultrasonography and have expedited surgical repair. The efficacy in determination of rotator cuff tears will also increase.

**Methods:**

Patients were retrospectively reviewed from January 2007 to December 2012. They were divided into 2 groups: Ultrasound (-) group and the Ultrasound (+) group. Age, gender, wait time from outpatient department (OPD) visit to MRI exam, MRI exam to operation (OP), and OPD visit to OP, patient number for MRI exam, and number of patients who finally had rotator cuff repair within two groups were compared.

**Results:**

The wait time of OPD visit to OP and MRI to OP in patients who received shoulder ultrasonography was significantly less than that in patients did not receive shoulder ultrasonography screening. Only 23.8% of the patients with a suspected rotator cuff injury undergone arthroscopic rotator cuff repair before ultrasonography was applied as a screening tool. The percentage increased to 53.6% after orthopedic surgeon started using ultrasonography as a screening tool for rotator cuff tears.

**Conclusions:**

Office-based shoulder ultrasound examination can reduce the wait time for a shoulder MRI. The efficacy of determination of rotator cuff tears will also increase after the introduction of shoulder ultrasonography.

## Background

Rotator cuff tears are a common cause of debilitating pain, reduced shoulder function, and weakness. This condition affects 40% or more of patients older than 60 years and is encountered in about 50% of patients over 70 years [1, 2]. Forty percent of rotator tears, if left untreated, increase in size, while none of these cases show a decrease in the size of the tear or spontaneous healing [3]. According to Moosmayer et al. [4], out of 50 subjects with an asymptomatic rotator cuff tear at the study start, 18 developed symptoms at a mean time of 18 months. The progression of tear size and deterioration of muscle quality occurred to a greater extent in the group that had developed symptoms. Shoulder stiffness frequently accompanies rotator cuff tears, with a reported incidence of up to more than 40% [5]. Traditionally, when a patient presents with a rotator cuff tear and a concomitant stiff shoulder, the stiffness is treated first through non-operative treatment until the patient regains passive range of motion (ROM) and surgery for rotator cuff repair is subsequently performed. However, this staged treatment may be insufficient, especially in patients with diabetes [6], and the rotator cuff tear may extend during the treatment of the stiffness because of the stretching and the delay of surgery. Some recent studies suggested simultaneous treatment of the rotator cuff tear and stiffness [7, 8]. Performing physical examinations in patients with stiff shoulder, however, is difficult because of pain and deficient shoulder ROM. Imaging studies, therefore, are suggested in combination with physical tests to reduce the uncertainty about diagnosing rotator cuff disease [9].

Magnetic resonance imaging (MRI) provides an anatomic picture, demonstrates the quality of rotator cuff muscles, and is very commonly used in clinical practice for detection of rotator cuff tears or shoulder stiffness. However, the cost of MRI examinations is far beyond that of an ultrasonography exam. Shoulder ultrasonography performed by a technician and interpreted by a radiologist with expertise has been shown to be accurate in detecting full-thickness and partial-thickness tears of the rotator cuff [10]. The sensitivity and specificity of ultrasonography for the diagnosis of symptomatic full-thickness rotator cuff tear were reported to range from 91 to 100% and 85 to 86%, respectively [11, 12]. The technique also provides bilateral information without being affected by the presence of intra-osseous hardware, is better tolerated, and allows the patient to view real-time information with immediate results. It is also less expensive than MRI. If the integrity of the rotator cuff is confirmed by shoulder ultrasonography, orthopedic surgeons and patients can be more confident of achieving successful results with non-operative treatment in patients with stiff shoulder without rotator cuff tear. Therefore, there will be no immediate indications to arrange MRI examinations for this group of patients. If patients with rotator cuff tears with or without shoulder stiffness are screened and diagnosed by shoulder ultrasonography at the orthopedic office, the subsequent surgical treatment can be carried on, avoiding progression of the rotator cuff tear because of the delay caused by a long wait time of MRI exams.

However, in comparison with other modalities such as MRI, the use of ultrasonography for the diagnosis of rotator cuff disease in authors’ hospital has achieved only limited acceptance among orthopedic doctors because of the uncertainty over the accuracy of this modality, which is not performed by the orthopedic surgeons themselves. We hypothesize that a well-trained orthopedic surgeon can utilize shoulder ultrasonography in conjunction with physical examinations, patient history, and a review of shoulder radiographs to accurately diagnose the rotator cuff pathology, thus allowing the provision of a so-called one-stop clinic, saving time and hospital visits, and potentially offering cost and time savings for patients who actually have rotator cuff tears. The waiting time of MRI exams should decrease once orthopedic surgeons start utilizing shoulder ultrasonography because patients who really have a rotator cuff tear will be notified as soon as they received ultrasonography screen. These patients could be arranged MRI exam with priority because they do have a rotator cuff tear. The positive detection rate in determining rotator cuff tears through MRI in all patients suspected to have this condition should increase after orthopedic surgeons start utilizing shoulder ultrasonography.

## Methods

### Patient review

This study was approved by the ethics committee of authors’ hospital (IRB 102-0160B). A retrospective review of consecutive patients who had visited AC’s OPD and were suspected to have rotator cuff tears was collected. Patient information was anonymized and de-identified prior to analysis. Written informed consent was given by participants for their clinical records to be used in this study at the first OPD according to author’s hospital’s regulations. Patients who were suspected to have rotator cuff tears were arranged shoulder MRI exam at OPD or a day before surgery by a single orthopedic surgeon (AC). Patients presenting to clinic with previously obtained MRI images from other hospitals were excluded from this study because it was difficult to measure the wait time from MRI exam done elsewhere to index surgery at author’s hospital. Patients were divided into two groups: ultrasound (-) and ultrasound (+). Patients from the ultrasound (-) group (from January 2007 to December 2010) were those who had arranged MRI exam at OPD without any prior shoulder ultrasonography screening. Patients from the ultrasound (+) group (from January 2011 to December 2012) were those who had arranged MRI exam at OPD with prior shoulder ultrasonography screening on the same day. Patients in the ultrasound (-) group underwent MRI exams without prior shoulder ultrasonography screening because radiologists in our hospital were not available to perform shoulder ultrasonography for orthopedics at that time. After January 2011, another orthopedic staff member (CC), who also majored in shoulder surgery, was trained by an experienced radiologist in our hospital, and he was responsible for the shoulder ultrasonography screening in any patient who was suspected to have rotator cuff tears, especially in those with concomitant stiff shoulder that made it difficult to perform a physical examination. Since then, patients who were suspected to have rotator cuff tears in AC’s clinic were referred to the orthopedic examination room on the same day and underwent shoulder ultrasonography screening performed by CC. If there were no signs of rotator cuff tears in shoulder ultrasonography, a rehabilitation program was taught to the patient first. If a rotator cuff tear was suspected by the shoulder ultrasonography screening, the MRI exam was arranged in the same OPD visit or one day before arthroscopic rotator cuff repair if the patient decided to receive surgical repair.

Demographic data of all the patients are shown in Table 1, comparing the ultrasound (-) and ultrasound (+) groups for age, gender, number of patients who received MRI exams, final rotator cuff repair, and wait time between the first outpatient visit (OPD) to MRI exam (arranged when patients were suspected to have rotator cuff tears), MRI exam to operation day (OP), and OPD to OP.

**Table 1:** Demographic data of patients in the ultrasound (-) and ultrasound (+) groups and wait time from OPD to MRI, MRI to OP, and OPD to OP

			Gender	days		
	Year	Age	Male/female	OPD to MRI	MRI to OP	OPD to OP
Ultrasound (-) *n* = 50	2007	57.6 ± 7.9	9/7	51.3 ± 17.5	26.9 ± 19.2	78.2 ± 30.0
	2008	59.2 ± 10.3	6/7	29.8 ± 16.0	67 ± 71.6	96.8 ± 66.8
	2009	55.6 ± 10.8	3/4	23.1 ± 22.6	49.3 ± 41.8	72.4 ± 45.6
	2010	61.1 ± 7.5	8/6	17.9 ± 13.8	35.2 ± 34.7	53.1 ± 35.8
	Overall	58.7 ± 8.8	26/24	32.4 ± 21.4	42.8 ± 46.5	75.2 ± 47.2
Ultrasound (-) *n* = 67	2011	56.7 ± 10.5	11/10	32.7 ± 17.7	26 ± 26.4	58.7 ± 26.8
	2012	61.3 ± 10.8	23/23	23.5 ± 11.9	6.3 ± 16.0	29.8 ± 22.1
	Overall	59.9 ± 9.5	34/33	26.4 ± 14.5	12.4 ± 21.7	38.8 ± 27.1

### Training for ultrasound examination

The American Institute of Ultrasound in Medicine has published training guidelines for medical practitioners who are not radiologists to direct development of proficiency in musculoskeletal ultrasound. This publication suggests that a clinician must have undergone training under the supervision of a qualified musculoskeletal ultrasonographer to develop proficiency in musculoskeletal ultrasound. Due to the policy of our institute, experienced radiologists are no longer available for shoulder ultrasonography for orthopedic surgeons. Therefore, CC, who also majored in shoulder arthroscopic surgery, has been responsible for shoulder ultrasonography arranged by orthopedic surgeons in the Orthopedic Sports Medicine Department in our Institute since January 2011. Before that, he had been well trained according to aforementioned guidelines and had cooperated with an experienced radiologist who once majored in shoulder ultrasonography.

### Imaging techniques

**Fig. 1:**
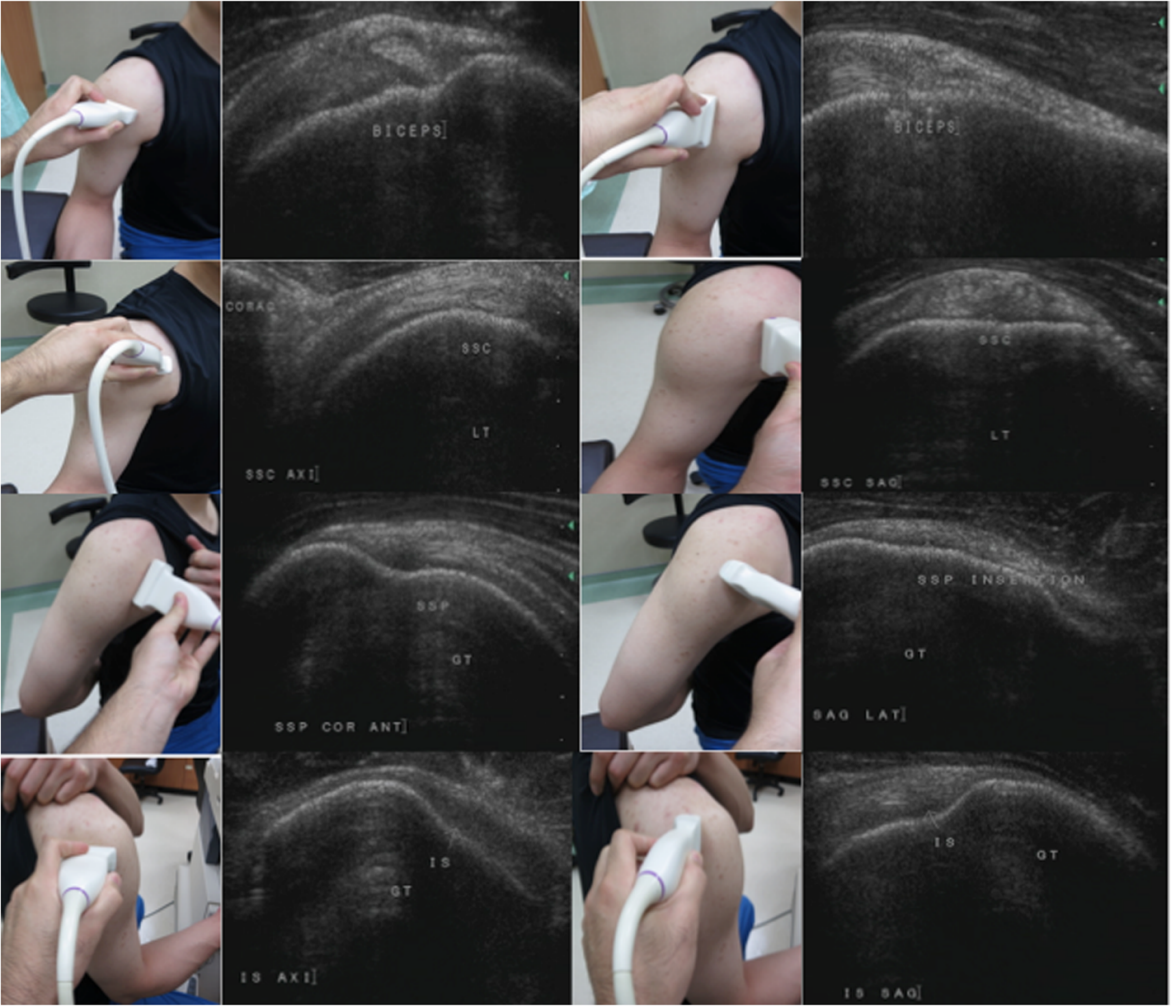
Positions in shoulder ultrasonography examination

Before performing shoulder ultrasonography, the duration and extent of subjective pain and the physical examination (including painful arc, Hawkins, and empty-can tests) were checked by CC. All ultrasonography results were obtained in a real-time fashion by a Phillips HDI 5000 scanner or Phillips EPIQ 5 scanner and a variable high-frequency linear-array transducer (7.5–10 MHz). All the patients had undergone a standardized bilateral shoulder ultrasonography performed by CC. The shoulder ultrasonography examination was performed with the patient seated on a stool and the doctor sat behind the patient. First, the bicep tendon was examined in the transverse and sagittal planes from the level of the acromion inferiorly to the point where the tendon merged with the bicep muscle. Images of the supraspinatus tendon were made with the extended shoulder, the flexed elbow, and the hand placed on the iliac wing. This position was considered necessary to expose as much as possible the supraspinatus tendon from under the acromion. The transducer was oriented parallel to the tendon (approximately 45° between the coronal and sagittal planes) in order to visualize the fibers in a longitudinal plane, and it was anteriorly to posteriorly moved in order to visualize the supraspinatus and infraspinatus tendons. The transducer was then 90° rotated in order to examine the tendons in the transverse plane, as shown in Fig. 1.

A full thickness rotator cuff tear was defined when the rotator cuff could not be visualized because of a complete avulsion and retraction under the acromion or when there was a focal defect in the rotator cuff created by a variable degree of retraction of the torn tendon ends. A partial-thickness tear was defined when a distinct hypo-echoic or mixed hyper-echoic and hypo-echoic defect was visualized in both the longitudinal and the transverse planes at the deep articular side of the rotator cuff (an articular side partial-thickness tear) or when there was a minimal flattening of the bursal side of the rotator cuff (a bursal side partial-thickness tear). A thinned cuff or one with a subtle concave contour was considered to be intact in the absence of a focal defect [12].

The extent of the rotator cuff tear was determined with transverse measurements. If the tear extended 1.5 cm posteriorly from the intra-articular portion of the biceps tendon, it was recorded as involving only the supraspinatus tendon, whereas if it extended >1.5 to 3.0 cm, it was recorded as involving both the supraspinatus and the infraspinatus tendon. The teres minor tendon was not evaluated [12–16].

MRI scans were performed in all patients with suspected rotator cuff tears who did not undergo a shoulder ultrasonography screening before January 2011 and in all patients with rotator cuff tears screened by shoulder ultrasonography as office-based exam after January 2011. All examinations were performed in a closed 1.5-T magnet with a field of view from 14 to 16 cm and the use of T1 and T2-weighted image sequences in sagittal, coronal oblique, and axial planes. Intra-articular gadolinium was sometimes administered, especially when partial-thickness tear of rotator cuff or concomitant labrum lesions were suspected. The criterion for a full-thickness rotator cuff tear was a fluid-filled gap in the tendon noted on the T2-weighted sagittal or coronal oblique images. A partial-thickness tear was defined as an increase in the signal noted on the T1-weighted images, with a brighter signal on the T2-weighted paired image. The total size in the anterior-posterior and medial-lateral dimensions was measured in centimeters using the MRI scale noted on the images [17].

### Indications for surgery

The indications for operation (OP) included shoulder pain of more than 3 months’ duration and a lack of a response to non-operative treatment including physical therapy, non-steroidal anti-inflammatory medications, and at least three cortisone injections. Patients with a full-thickness tear who had a recent traumatic injury (such as direct contusion or shoulder dislocation) diagnosed through shoulder ultrasonography or MRI were offered the option of an operation within less than 3 months.

### Statistical methods

In the present study, we categorized patients into two groups; patients recruited from Jan 2007 to December 2010 did not receive ultrasound examination (ultrasound (-) group) whereas patients recruited from January 2011 to December 2012 were further examined with ultrasound (ultrasound (+) group). Critically, the independent *t* test was conducted to test the difference of wait time between OPD to MRI, MRI to OP, and OPD to OP for patients with and without ultrasound examination. Ninety-five percent confidence intervals were calculated for each of the proportions with the use of a normal approximation method. Violations of Levene’s test of homogeneity of variance were corrected. Moreover, the Chi-square test for the positive rate of final OP was also performed.

## Results

**Fig. 2:**
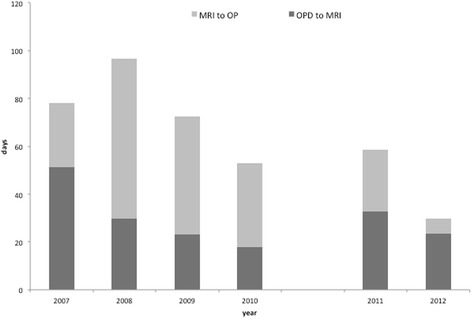
Wait time between the first outpatient clinic OPD visits to MRI exams (OPD to MRI), MRI exams to operation (MRI to OP), and OPD to OP

The ultrasound (-) group included 50 patients (26 males, 24 females) that had received arthroscopic rotator cuff repair from January 2007 to December 2010. Their mean age was 58.7 ± 8.8 years (range, 39–73 years). In the ultrasound (+) group, there were 67 patients (34 male, 33 female) that had received arthroscopic rotator cuff repair from January 2011 to December 2012. Their mean age was 59.9 ± 9.5 years (range, 28–84 years). Patients in the ultrasound (-) group had waited a mean of 32.38 ± 21.4 days from OPD to MRI exam, 42.78 ± 46.5 days from MRI to OP, and 75.16 ± 47.2 days from OPD to OP. Patients in the ultrasound (+) group had waited 26.4 ± 14.5 days from OPD to MRI, 12.4 ± 21.7 days from MRI to OP, and 38.8 ± 27.1 days from OPD to OP, which are shown in Table 1 and Fig. 2. The independent *t* test showed that there were significant differences between groups in the wait time from MRI to OP (*t* (1,115) = 4.28, *p* < .01) and OPD to OP (*t* (1115) = 4.88, *p* < .01), whereas there was no difference in the wait time of OPD to MRI (*t* (1115) = 1.71, *p* = .09).

Two hundred ten patients received an MRI exam in the ultrasound (-) group, while only 50 of these patients underwent an operation eventually. Only 23.8% of the patients who had been suspected to have rotator cuff injury proceeded to undergo an arthroscopic rotator cuff repair. In the ultrasound (+) group, 175 patients who were suspected to have rotator cuff tears diagnosed through history taking and physical examination were arranged shoulder ultrasonography. Among them, 125 were diagnosed rotator cuff tears from ultrasonography and MRI were arranged. One hundred thirteen rotator cuff tears were found in MRI exam. After discussion about the pros and cons of surgical repair, 67 patients had received surgery and all of them had rotator cuff tears confirmed through arthroscopy. The positive rates of patients who received surgery among the patients performed MRI in the ultrasound (+) group was 53.6% (Table 2). The sensitivity of ultrasonography, MRI, and arthroscopy to diagnose rotator cuff tears were 90, 100, and 100%, respectively.

**Table 2:** The positive rate in patients receiving surgery among patients undergoing MRI in the ultrasound (-) and ultrasound (+) groups

	*n* for MRI screening	*n* for OP	Positive rate (%)
Ultrasound (-)/2007–2010	210	50	23.80
ultrasound (+)/2011–2012	125	67	53.60

### Patient demonstration

#### Case 1

**Fig. 3:**
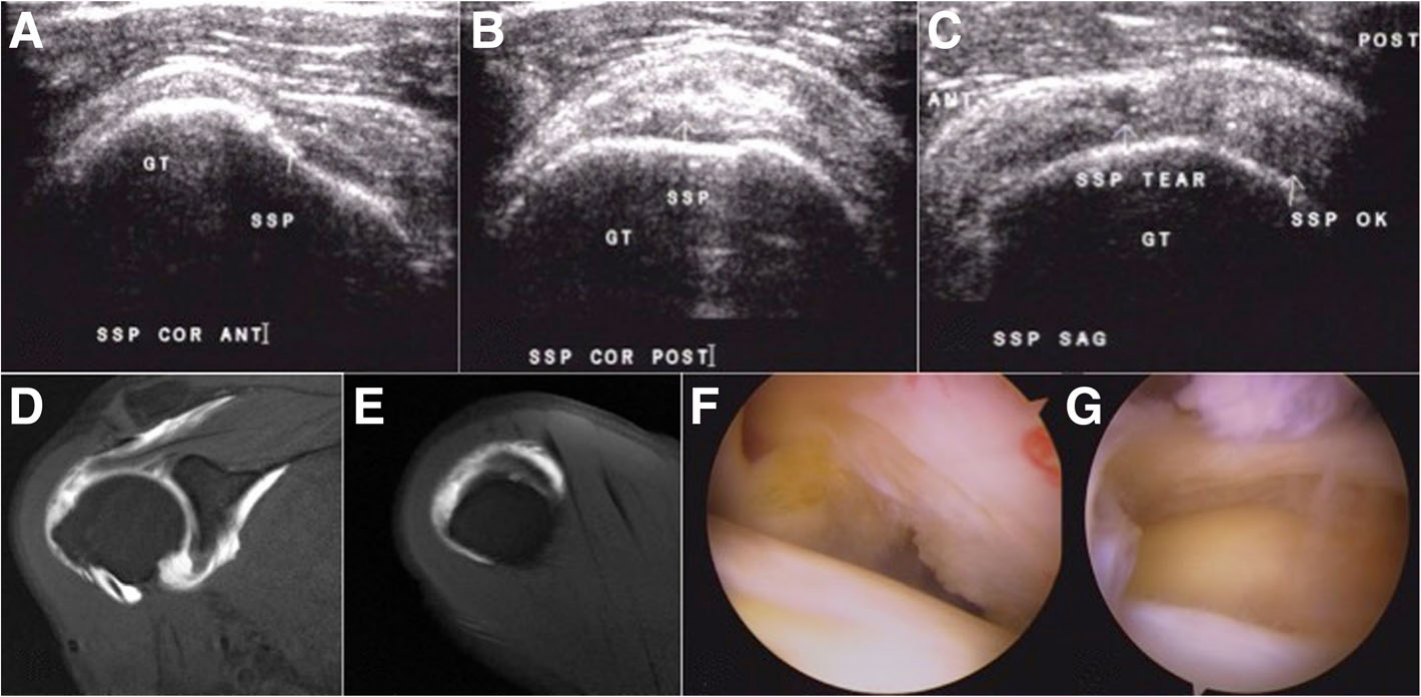
Case 1. A 60-year-old male patient with a full-thickness tear of the anterior part of the supraspinatus tendon. **a** Coronal ultrasonographic view of the anterior part of the supraspinatus. **b** Coronal ultrasonographic view of the posterior part of the supraspinatus. **c** Sagittal ultrasonographic view of the lateral part of the supraspinatus. **d** Coronal MRI view of the anterior part of the supraspinatus. **e** Sagittal view of MRI of the lateral part of the supraspinatus. **f** Intra-articular view of the anterior part of the supraspinatus tendon tear. **g** Subacromial view of the anterior part of the supraspinatus tendon tear

A 60-year-old male patient without any history of recent trauma had been experiencing disabling pain in the right shoulder for 5 months. The pain progressed at night, especially when he rested on his right shoulder. The weakness of the right shoulder was noted when he lifted objects that weighed more than 5 kg. There were neither limitations in range of motion nor atrophy of the musculature of his right shoulder. The empty-can test yielded positive results, but there was no dropping sign and external rotation lag in his right shoulder. Ultrasonography at the same OPD revealed a full-thickness tear of the anterior part of his supraspinatus tendon (Fig. 3a–c). After a thorough discussion about the pros and cons of surgical repair of the torn supraspinatus, the patient underwent an MRI exam the day before surgery to determine the extent of supraspinatus tear and its associated injuries (Fig. 3d–e). The arthroscopic view of the supraspinatus tear of the patient is shown in Fig. 3f–g.

#### Case 2

**Fig. 4:**
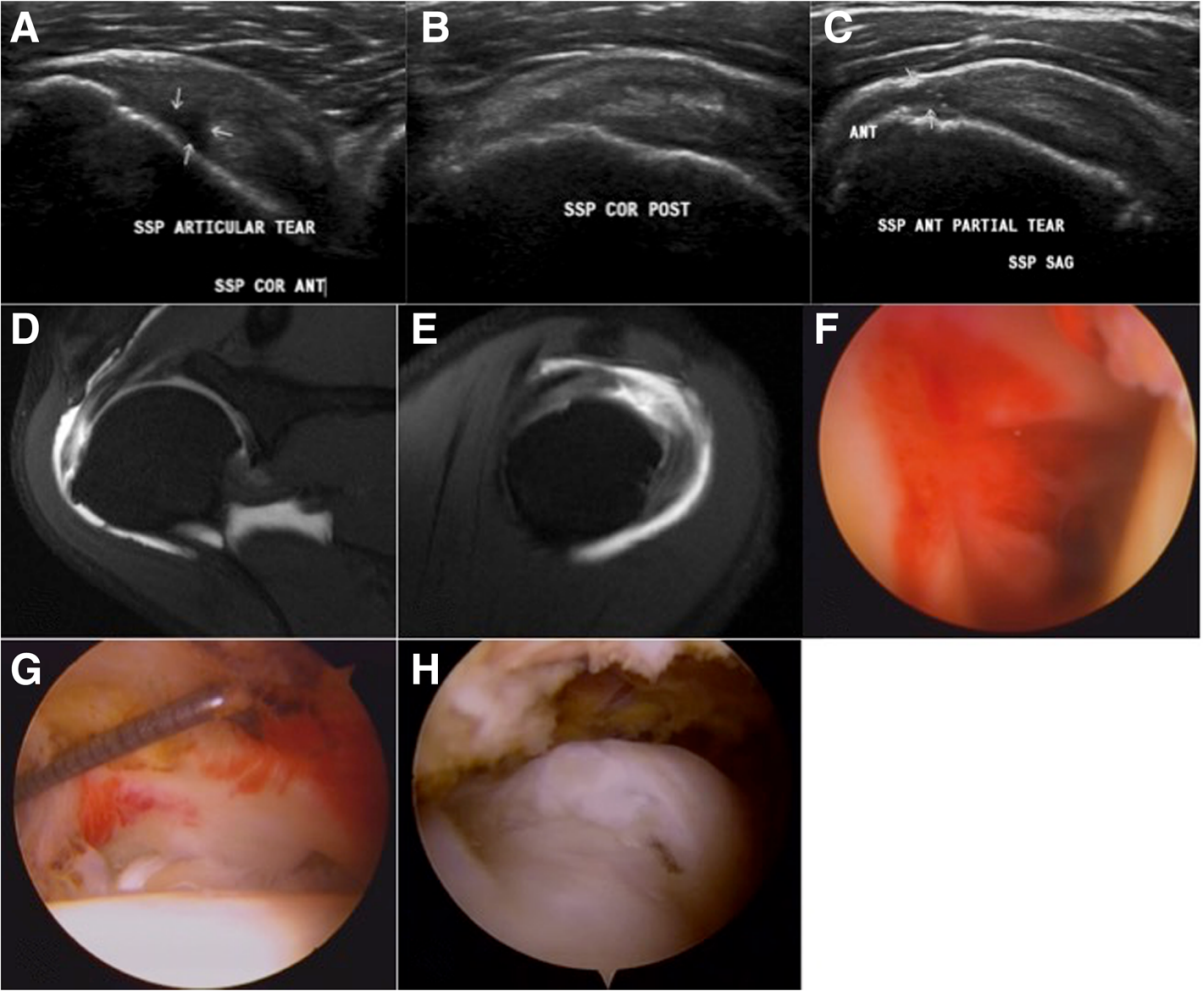
Case 2. A 52-year-old male patient with diabetes had right shoulder stiffness and a near full-thickness rotator cuff tear. **a** Coronal ultrasonographic view of the anterior part of the supraspinatus. **b** Coronal ultrasonographic view of the posterior part of the supraspinatus. **c** Sagittal ultrasonographic view of the lateral part of the supraspinatus. **d** Coronal view of MRI of the anterior part of the supraspinatus. **e** Sagittal view of MRI of the lateral part of the supraspinatus. **f** Intra-articular view of the shoulder adhesive capsulitis. **g** Intra-articular view of the anterior part of the supraspinatus tendon near the full-thickness tear. **h** Subacromial view of the anterior part of the supraspinatus tendon near the full-thickness tear

A 52-year-old male patient with diabetes experienced disabling right shoulder pain and stiffness for 6 months. The pain progressed especially at night. Physical examination showed a forward elevation of about 120°, abduction of about 120°, external rotation by the side of about 30°, and internal rotation to the sacral area. The patient found it difficult to perform the empty-can test because of the severe pain and could not perform the belly press test and lift-off test. Ultrasonography at the same OPD revealed a near-full thickness tear of the anterior part of his supraspinatus tendon (Fig. 4a–c). An MR arthrogram exam was arranged as shown in Fig. 4d–e. An arthroscopic view of the adhesive capsulitis combined with supraspinatus tear of the patient was shown in Fig. 4f–h.

## Discussion

Rotator cuff tendon tears account for more than 4.5 million physician visits per year, and over 250,000 rotator cuff repair surgeries are annually performed in the USA [2]. Such conditions are, however, occasionally asymptomatic until they lead to anatomic deterioration and decrease the muscle quality, which are correlated to the development of symptoms [18]. Currently, no single test is alone sufficient to diagnose rotator cuff disease [9]. MRI provides an anatomic picture, demonstrates the quality of rotator cuff muscles and the degree of tendon retraction, and shows other eventual intra-articular and extra-articular pathologies [11]. However, MRI examinations are expensive to perform and motion artifacts cannot be avoided, especially when the patient with parkinsonism, which can be extremely problematic in restless patients or in those who suffer from claustrophobia [19]. Arthrography is also a powerful tool for detecting rotator cuff disease, but the disadvantages of this technique include its invasive nature and the great number of false-positive results following rotator cuff repair because of a non-watertight closure in patients during follow-up [20].

Ultrasonography of the shoulder had been recently employed as a screening tool because it is simple, quick, affordable, and provides an immediate imaging method as an adjunct to clinical evaluation and a high rate in detection of full-thickness rotator cuff tears [21]. Yamamoto et al. performed ultrasound screening for rotator cuff tears in 683 residents of a Japanese mountain village and revealed that 283 shoulders in 211 individuals between 34 and 87 years of age had full-thickness rotator cuff tears [22]. Ultrasonography is cheaper and quicker than MRI and it is as accurate as MRI in the detection of rotator cuff tears [23, 24]. High-resolution ultrasonography is believed to have 100% sensitivity, 85% specificity, and 96% accuracy in detecting full-thickness rotator cuff tears [12]. In a study of 61 patients, Brenneke and Morgan found that ultrasonography had a sensitivity of 95% and a specificity of 93% for the detection of full-thickness tears [25]. Thus, ultrasonography may help to screen patients effectively prior to more advanced imaging methods in some cases [26].

Shoulder ultrasonography is comparable with MRI in detecting the size of cuff tears. According to Iannotti et al., the sensitivity of ultrasonography for detecting the tear size in the anteroposterior dimension was 86% (95% confidence interval, 71–95%) and that of MRI was 93% (95% confidence interval, 81–99%). The sensitivity of ultrasonography for detecting the tear size in the medial-lateral dimension was 83% (95% confidence interval, 69–93%) and that of magnetic resonance imaging was 88% (95% confidence interval, 74–96%) [17].

In fact, shoulder ultrasonography may be better than MRI because of its capability to evaluate the cuff muscles globally from their insertions to their origins in real-time fashion since MRI is a static examination [19]. Another advantage of shoulder ultrasonography is that it is office-based and can be performed at the time of the patient’s clinical evaluation, so there is no need to schedule another appointment for the test, which is very convenient for the patient [17]. It is also faster in comparison with MRI, with the average time per ultrasound examination being less than 10 min when performed by experienced examiners [27]. Patients with shoulder pain prefer ultrasound over MRI, and they are more willing to repeat an ultrasound examination according to Middleton et al. [28].

The inherent limitation of the usage of shoulder ultrasonography in detecting rotator cuff tears is its high-operator dependence, and the accuracy of ultrasound as a diagnostic modality may vary from institution to institution, depending on the level of expertise of the musculoskeletal radiologist [17, 29]. Ultrasonography relies much more on the experience and skills of the operator than MRI and CT do, and it has a long, steep learning curve [19]. However, it is thought nowadays to be an extension of the physical examination and the clinical evaluation of the shoulder at the time of the patient’s office visit. Orthopedic surgeons have started performing shoulder ultrasonography by themselves, and several studies of orthopedic surgeons with substantial ultrasound experience have shown that their assessment of the rotator cuff is comparable with that done by musculoskeletal radiologists [30–32]. Murphy et al. had proposed an independent learning protocol for orthopedic surgeons performing shoulder ultrasound in identifying full-thickness rotator cuff tears and found that the agreement among these clinicians and the radiologist was equally high [26]. According to their results, a first-year orthopedic resident agreed with radiologist about the findings in 85% of the eighty-two shoulders that were scanned by both the resident and a radiologist, whereas the specialist shoulder surgeon agreed with the radiologist about the findings in 83% of the seventy-eight shoulders that were scanned by both the specialist and a radiologist. Sixty-three shoulders were scanned by both the specialist shoulder surgeon and the first-year orthopedic resident. They agreed about the presence or absence of a full-thickness rotator cuff tear in 86% of the shoulders. They suggested that orthopedic surgeons with varying levels of clinical experience could become competent in shoulder ultrasound in a relatively short period of time by following an independent learning program.

Since January 2011, CC, who majored in shoulder surgery, was responsible for office-based shoulder ultrasonography screening for all patients with suspected rotator cuff tears in a single doctor’s clinic (AC) on the same day. Patients diagnosed with rotator cuff tears by shoulder ultrasonography underwent discussions about possible surgical treatment if symptoms were not resolved in more than 3 months since the initial episode of painful disability. MRI was arranged right away on the same day if there were no contraindications such as claustrophobia. These patients would be marked to have rotator cuff tears when referring to radiology department to receive MRI exam, and they would get priority in the waiting list since they already had preliminary results of cuff condition by ultrasound screening. If patients asked for expedited rotator cuff repair, they would be admitted one day before surgery to undergo a pre-arranged MRI exam without waiting for MRI with other patients. The change in the protocol for identifying rotator cuff tears before and after shoulder ultrasonography introduction contributed a significantly decreased waiting time from MRI to OP and OPD to OP. This phenomenon implied the usage of shoulder ultrasonography changed the diagnostic behavior of orthopedic surgeons and also decreased the wait time for patients who really needed and wanted surgery. The positive rate for patients who actually received index surgery increased from 23.8 to 53.6% after shoulder ultrasonography was performed. Before shoulder ultrasonography performed by orthopedic surgeons, only about one-fourth of the patients with suspected rotator cuff tears proceeded to the final surgery. After the beginning of ultrasonography as a screening tool, the patients who actually had rotator cuff tears were detected sooner than before and a greater proportion of them underwent surgical repair, instead of struggling in the long waiting list of MRI exam. The prolonged waiting time for MRI exam might place patients at risk of future complications such as tear progression. Those who did not have rotator cuff tears detected by shoulder ultrasonography were referred to the rehabilitation department first, instead of undergoing an MRI exam. This approach decreased the amount of unnecessary MRI exams, especially for those patients who had difficulties in performing physical examination such as patients with severe stiff shoulder. Thus, appropriate treatment (e.g., surgical repair of torn rotator cuff) could begin immediately to avoid clinical progression of patients who really had rotator cuff tears.

There are still some limitations in this study. First, this was a retrospective study to determine how shoulder ultrasonography changes the orthopedic surgeon’s behavior in detecting rotator cuff tears. However, it is difficult to conduct a prospective study because our hospital is a general hospital with many patients who have other diseases than rotator cuff tears and need an MRI exam elsewhere. Orthopedic surgeons can only notify radiology department about patients who had been previously screened for rotator cuff tears. Therefore, if the patients ask for expedited surgical treatment, we will arrange MRI 1 day before surgery instead of letting them wait for an MRI exam with patients of other diseases. No patients in our retrospective study had canceled operation after the MRI exam during admission or had their treatment plan changed because our high-detection rate of rotator cuff tears through shoulder ultrasonography at outpatient clinic. The other concern is that with a prospective study, we could hardly see the change of behavior of orthopedic surgeons in detecting rotator cuff tears. Before ultrasonography screening, all patients suspected to have rotator cuff tears were arranged MRI exam, but some of them did not really have rotator cuff tears. By ultrasonography screening, we could locate those who really had rotator cuff tears. Second, the inherent limitation of ultrasonography is its operator dependence. According to Murphy et al. [26], orthopedic surgeons with varying levels of clinical experience can become competent in shoulder ultrasound in a relatively short period of time by following an independent learning program. The orthopedic doctor performing shoulder ultrasonography (CC) is also majored in shoulder arthroscopy as everyday surgical practice, which will improve the ability of assessment of the rotator cuff tears. Third, patients who were diagnosed with rotator cuff tears did not always receive surgical repair. They may have undergone other kinds of treatment, even if surgery was indicated and discussed. If patients asked for surgery, they frequently scheduled surgery around their life events and subsequently surgical scheduling can be delayed by weeks or even months when earlier spots are available. This variable is very difficult to be controlled, especially in a retrospective study and the non-emergent nature of a rotator cuff tear. Hence, we enrolled only patients receiving surgical repair instead of all the patients who were diagnosed to have rotator cuff tears, in order to see the difference in wait time in different treatment stages. Fourth, the results can only be applied in hospitals in which no personnel are responsible for shoulder ultrasonography before. If radiologists, or even young orthopedic residents under adequate training, are available for shoulder ultrasonography, the significant changes in protocol of treating rotator cuff tears may not be found. Therefore, the situation can only be observed in several years before and after shoulder ultrasonography introduction. Fifth, the result can only be applied to a general hospital, where patients have to compete for the limited space available of MRI exams with other patients with different kinds of diseases. In clinics specific for sports medicine or with plenty of room for MRI exams, patients may undergo MRI exam right away instead of waiting for such a long time once rotator cuff tear is suspected.

The strength of this study is that all the pros/cons, indications and contraindications of rotator cuff repair are discussed with patients by the same orthopedic surgeon (AC). This can eliminate the selection bias regarding surgical preference by different doctors.

## Conclusions

Office-based shoulder ultrasound examination can be used in conjunction with the patient’s clinical history and physical examination and provides important information regarding rotator cuff condition. It reduces wait time from first outpatient visit to final surgery and MRI to final surgery and in patients with rotator cuff tears. Higher positive detection rates regarding rotator cuff tears were found among patients who received shoulder MRI exam. Improved diagnostic accuracy with better clinical correlation can not only facilitate subsequent treatment planning but also lessen the overwhelmingly tight schedule of screening MRI for equivocal cases.

## Abbreviations

MRI: Magnetic resonance imaging
OP: Operation
OPD: Outpatient department
ROM: Range of motion
